# Systematic review of intraoperative radiation therapy for head and neck cancer

**DOI:** 10.3332/ecancer.2022.1488

**Published:** 2022-12-12

**Authors:** Cesar Vincent L Villafuerte, Aveline Marie D Ylananb, Harroun Valdimir T Wong, Johanna Patricia A Cañal, Edilberto Joaquin V Fragante

**Affiliations:** Division of Radiation Oncology, Department of Radiology, University of the Philippines Manila - Philippine General Hospital, Manila 1000, Philippines; a https://orcid.org/0000-0002-4024-6262; b https://orcid.org/0000-0003-0795-4221; c https://orcid.org/0000-0003-1973-1535; d https://orcid.org/0000-0001-5407-1478; e https://orcid.org/0000-0001-6018-4495

**Keywords:** head and neck malignancies, intraoperative radiation therapy, review

## Abstract

Multidisciplinary treatments with surgery, radiation therapy, and chemotherapy are the cornerstones in the management of locally advanced head and neck malignancies. In most cases, radiation is delivered via external beam radiation therapy (EBRT). Intraoperative radiation therapy (IORT), on the other hand, is the delivery of precise doses of radiation to selected target volumes within the exposed surgical field while at the operating room. Most studies on its use on head and neck cancers are limited to single-institutional retrospective case series. We performed a systematic review to consolidate the existing literature on IORT for head and neck malignancies. Fifty-two studies representing a mixed population of 2,389 patients were included in this review. IORT via electrons (intraoperative electron radiation therapy), brachytherapy (intraoperative high dose-rate brachytherapy) or photons was administered in numerous settings, but most commonly as part of a reirradiation regimen following salvage surgery for recurrent tumours. Often, additional EBRT was also planned postoperatively. This review illustrates that IORT is a promising treatment modality in head and neck cancer. Multiple single-institutional studies spanning several decades have demonstrated benefit in terms of local control with reasonable toxicity. However, randomised trials comparing it with current standards of care are still needed.

## Highlights

- Most publications on head and neck intraoperative radiation therapy (IORT) are single institutional and retrospective
- IORT is most often used with salvage surgery in previously irradiated cases
- IORT provides additional local control, especially after margin-negative resection
- IORT demonstrated reasonable acute and late toxicity rates
- Randomised trials comparing it with current standards of care are still needed

## Background

Multidisciplinary and multimodality treatments are the cornerstone in the management of locally advanced head and neck malignancies. The primary modalities of treatment are surgery, external beam radiation therapy (EBRT) and chemotherapy [1, 2]. Survival however remains low both in the primary [3] and recurrent setting [4], leading to opportunities to explore additional modes of treatment in order to achieve better outcomes.

Intraoperative radiation therapy (IORT) is the delivery of precise doses of radiation to selected target volumes within an exposed surgical field in the operating room [5]. The advantages of this technique include: 1) removing the delay between surgery and adjuvant radiation therapy (RT), 2) increasing the certainty in target delineation due to immediate and directly observed localisation of treatment targets and 3) possibly decreasing toxicity through the ability to mobilise organs at risk away from the intended treatment region.

Currently, this treatment modality has been more extensively investigated in other cancer sites, while its use in head and neck malignancies has not been as well elucidated. Most studies on head and neck IORT are limited to single-institutional retrospective case series. Although prior systematic reviews have been done [6, 7], we aim to validate as well as update their findings with the latest available published reports in this evolving treatment modality. This systematic review aims to help consolidate the existing state of literature on IORT for head and neck malignancies which may guide intended users in the evidence-based use of this treatment modality.

The objective of this study is to describe the reported dose-volume prescriptions and the outcomes of IORT on local control (LC), locoregional control (LRC), disease free survival (DFS) and overall survival (OS), and acute and late toxicity in patients with head and neck cancer using a systematic review of published case series, comparative studies and clinical trials.

## Methods

**Figure 1.**
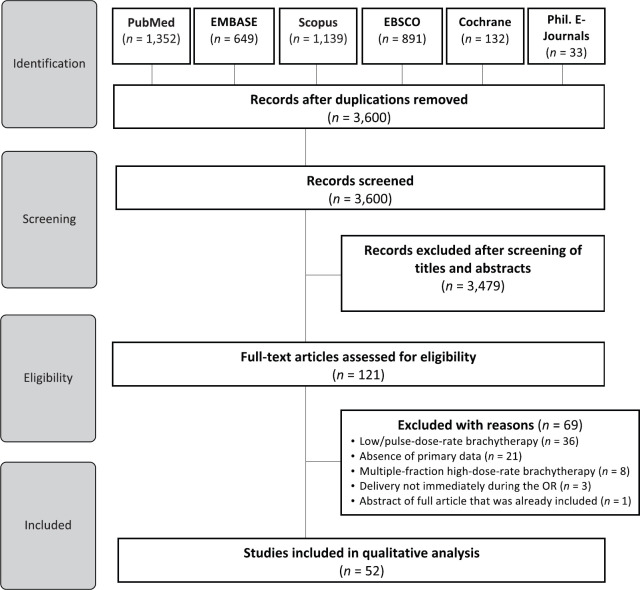
Preferred Reporting Items for Systematic Reviews and Meta-Analyses diagram.

Literature search as outlined in Figure 1 was done on 19 July 2021. Studies were limited to English language publications and included randomised and nonrandomised clinical trials, comparative studies, case series, previous systematic reviews and meta-analysis, on humans subjects who underwent IORT for head and neck malignancies with documented follow-up of at least 3 months after treatment to allow for the minimum duration of evaluation of acute as well as late toxicity. In addition to articles identified through database search, hand-search and snowballing of references from previously published reviews and initially selected individual studies were done to identify possible missed relevant articles for inclusion.

Search terms included: ALL FIELDS and Medical Subject Headings (MeSH) Terms for: ‘head and neck’ or ‘oral cavity’ or ‘pharynx’ or ‘nasopharynx’ or ‘oropharynx’ or ‘hypopharynx’ or ‘larynx’ or ‘paranasal sinus’ or ‘maxilla’ or ‘nasal cavity’ or ‘salivary gland’ or ‘thyroid’ or ‘skin’ or ‘orbit’ or ‘eye’ or ‘ear’ or ‘external auditory canal’ or ‘temporal’ or ‘skull base’ or ‘base of skull’ AND ‘Intraoperative Radiation Therapy’ or ‘intraoperative radiotherapy’ or ‘IORT’ or ‘intraoperative photon’ or ‘intraoperative electron’ ‘electron IORT’ or ‘photon IORT’ or ‘kV IORT’ or ‘IOERT’ or ‘intraoperative brachytherapy’ AND ‘cancer’ or ‘carcinoma’ or ‘malignancy’ or ‘neoplasm’ or ‘tumor’.

The articles retrieved from the database search were evaluated separately by authors AY and HW to determine if they met the pre-determined inclusion and exclusion criteria. Disagreements on inclusion or exclusion were resolved by consensus discussion together with authors CV, JC, and EF. Eligible primary literature studies remaining were included in this review paper.

### Internal validity and risk of bias assessment

Internal validity and risk of bias assessment was done using the National Institutes of Health National, Heart, Lung, and Blood Institute (NIH NHLBI) Quality Assessment Tools (https://www.nhlbi.nih.gov/health-topics/study-quality-assessment-tools) which allows for rating as good, fair or poor. Initial rating was done for each included study by CV, AY and HW. Final rating was obtained through consensus discussion meeting with all authors. Summary data extracted from the selected studies included: study design, duration of study, total number and demographics of the population, duration of follow-up, type of IORT technique and radiation dose regimen and volume prescription. In addition, effect measures included were: effect of IORT on local recurrence, locoregional recurrence, DFS, OS, acute toxicity and late toxicity in patients with head and neck cancer. Above review of studies were synthesised through tabulation of the selected studies.

This study was submitted to and approved by the institutional ethics review board.

## Results

### Literature search

We identified a total of 4,196 studies from the electronic database search. A total of 3,600 articles remained upon excluding duplicates. We excluded an additional 3,479 studies after assessing titles and abstracts. The full-texts of 121 articles were thereafter subjected to eligibility criteria. Finally, 52 studies were included in the qualitative analysis (Figure 1).

### Study characteristics, descriptions

The 52 studies were composed of observational cohorts and case series representing 2,389 patients. There was a male predilection, and the patients’ age ranged from 5 to 99 years old.

Head and neck subsites included: neck (20.4%), oropharynx (17.7%), oral cavity (14.4%), nasal cavity and paranasal sinuses (9.8%), hypopharynx (8.1%), parotid (8.1%), larynx (4.2%), other salivary glands (3.0%), skull base (2.9%), scalp or skin (2.5%), nasopharynx (1.4%), temporal bone (1.1%), orbit (0.9%), thyroid (0.3%) and others (4.96%). Majority of the cases were squamous cell carcinomas (SCCs) and locally advanced disease.

### Treatment setting

The authors were able to subdivide the 52 studies into two general headings – homogenous and heterogenous studies – with three subclassifications each (see Table 1 at https://doi.org/10.6084/m9.figshare.21676964.v1). The homogenous studies – where treatment settings were well defined – were subclassified as follows: 1) treatment in the primary setting using both IORT and EBRT (10 studies), 2) treatment in the recurrent setting treated with IORT alone (1 study) and 3) treatment in the recurrent setting using IORT alone in patients with a history of irradiation (4 studies).

Studies that included a mix of primary and recurrent cases, primary and reirradiation treatments, or IORT with and without EBRT were classified as heterogenous. The heterogenous studies were subclassified to: 1) treatment in the recurrent setting with or without prior irradiation using IORT and EBRT (6 studies), 2) treatment in the primary or recurrent setting with no prior irradiation using IORT and EBRT (4 studies) and 3) treatment in the primary or recurrent setting with or without prior irradiation using IORT with or without EBRT (27 studies).

## Homogenous studies

### a. Treatment in the primary setting using IORT + EBRT

Ten publications were observational cohorts of patients with SCC and no distant metastasis treated with IORT and EBRT in the primary setting.

Eight were from the Ohio State University series [8–14], which described the development of three intensification regimens (IRs) (IR-1, IR-2 and IR-3) for previously untreated, resectable, advanced SCC of the oral cavity, oropharynx or hypopharynx. The IRs all entailed neoadjuvant RT for 4 days followed by surgery with IORT on Day 4. Adjuvant EBRT ensued after, with different doses depending on the specific regimen.

In the final report by Schuller *et al* [12] on the institution’s 12-year experience, average compliance for all three regimens was 61% (75/123), LRC was 91% (112/123), systemic control was 86% (106/123) and overall disease specific survival was 73%. It was concluded that their approach was tolerable and provided good survival and control rates [14].

For the remaining two studies, Rutkowski *et al* [15] prescribed IORT of 5–7.5 Gy to 0.5 cm from the applicator surface with photons and Schmitt *et al* [16] used 20–25 Gy to D90% of the tumour bed with electrons. Majority of patients received additional EBRT of 50–65 Gy. LC rates were 100% and 60.5% at a median follow-up of 30 months and 24+ months in Rutkowski’s and Schmitt’s studies, respectively. Acute mucosal toxicity was noted in 14%–18.8% [15]. Late complications of laryngeal necrosis and laryngeal stenosis were seen in only two patients [9].

### b. Treatment in the recurrent setting with no previous RT using IORT alone

Only one study used IORT alone for previously unirradiated patients with recurrence. A retrospective case series from Memorial Sloan Kettering Cancer Center reported on the use of intraoperative high dose-rate brachytherapy (IOHDR) using Phosphorus 32 polymer radioactive films in Diffuse Recalcitrant Conjunctival Neoplasms. They delivered a median dose of 15 Gy to six patients with recurrent or residual disease after primary treatments for SCC, sebaceous carcinoma or lymphoma. At 24 months follow-up, they reported a recurrence free survival rate of 75%, improvement in visual acuity in 5 of the 6 patients, and only 1 severe adverse event of corneal ulcer and perforation [17].

### c. Treatment in the recurrent setting with previous RT using IORT alone

Among the four studies that used IORT alone for patients previously irradiated, three utilised electrons with doses of 10–25 Gy [18–20] and one utilised brachytherapy with doses of 10–15 Gy [21]. Outcomes varied with LC of 23.9%–61.5% and OS of 8%–54.9% [19]. It was also observed that LC was not improved in patients who had positive resection margins [18, 20]. Complications included wound healing problems, infection, fistula and necrosis [18–21]. Nag *et al* [19] reported severe complications in 16% of 38 patients. From these studies, we found that IORT alone to doses of 10–25 Gy in the salvage surgery setting is not enough to provide durable control, and that resection margin status is a significant factor in the outcomes of this patient population.

## Heterogenous studies

### a. Treatment in the recurrent setting with or without previous RT using IORT + EBRT

The studies which used IORT and EBRT in the recurrent setting with or without previous EBRT treatment had IORT dose ranging from 10–20 Gy for IOHDR [22–25] and 10–18 Gy for intraoperative electron radiation therapy (IOERT) [26, 27]. Previous EBRT doses received ranged from 24 to 80 Gy [23, 24] and additional EBRT doses given after IORT ranged from 30 to 84 Gy [25, 26]. It is important to note that among the six studies, more than 70% of patients had received prior EBRT and patients who received additional EBRT ranged from 13.5% to 55% [22, 27]. Outcomes were similar whether IOHDR or IOERT technique was utilised. LC, LRC, DFS and OS ranged from 57% to 82%, 51% to 77%, 37% to 61% and 34% to 53%, respectively [22–27]. The highest rates of toxicities were reported by Teckie *et al* [24], which include fibrosis (*n* = 17, 29%), trismus (*n* = 14, 24%), cranial nerve injury (*n* = 15, 26%), dysphagia (*n* = 23, 39%) and fistula/abscess (*n* = 9, 15%).

### b. Treatment in the primary or recurrent setting without previous RT using IORT + EBRT

There were four publications on patients treated in the primary or recurrent setting with no previous EBRT. These patients received 10–15 Gy IORT followed by adjuvant EBRT ranging from 50 to 60 Gy [28–31]. Good LC and LRC were seen ranging from 68% to 94.1% and 61% to 76.5%, respectively [28–31]. Notable toxicities reported were facial nerve paralysis (*n* = 4, 23.5%), necrosis/flap failure (*n* = 3, 23%) and fistula (*n* = 2, 8%) [29, 30].

### c. Treatment in the primary or recurrent setting with or without previous RT using IORT with or without EBRT

The remaining 27 studies included a heterogenous mix of patients with primary or recurrent disease, previously irradiated or unirradiated, who received IORT with or without additional EBRT. Majority of these studies utilised IORT doses up to 20 Gy and utilised electrons [32–56]. Among patients who received a previous course of EBRT, doses ranged from 26 Gy [32] to as high as 82 Gy [57] while additional EBRT doses after IORT ranged 10–70 Gy [32]. This set of studies has LC rates ranging from 25% to 100%, LRC rates from 11.2% to 80%, DFS rates from 9% to 70.3% and OS from 17% to 88% [32–58]. Due to the heterogeneity of the population included in these studies, large variations were observed in the outcomes, primarily coming from the studies using IOERT. Reported acute and late toxicity rates included wound infection (*n* = 20, 9.9%) [38], vascular [transient ischemic attack (TIA), carotid blowout, cardiac ischaemic events] (*n* = 23, 11.3%) [38], pain needing opiates (*n* = 16, 13.3%) [34], osteoradionecrosis (*n* = 4, 17.4%) [32] and mild nausea/vomiting (55.6%) [53].

### IORT type

Table 2 summarises the 52 studies discussed above grouped according to type of IORT used. Among the studies, the types of IORT used were electrons in 27, brachytherapy in 10, a mix of electrons or brachytherapy in 10 and photons in 5. The studies comprised 1,476, 445, 400 and 68 patients, respectively.

Majority of the patients who received IORT had recurrent disease (*n* = 1,376) compared to those who received IORT as part of their primary treatment (*n* = 793). Further, among the patients included in the selected studies, 1,095 (45.84%) patients had already received prior irradiation, while 935 (39.14%) patients had no previous irradiation, and 359 (15.03%) patients had unreported prior RT status. The average doses received by patients who had prior irradiation were 54.5 Gy, 61.6 Gy, and 34.1 Gy for studies using IOERT, IOHDR, or a mix of IOERT or IOHDR, respectively. IORT doses differed among the different modalities with an average dose of 15.6 Gy (range, 4-100 Gy) for studies on IOERT, 13.4 Gy (range, 5-20 Gy) for studies on IOHDR, 8.3 Gy (range, 7.5-20.0 Gy) for studies with a mix of IOERT or IOHDR, and 5.8 Gy (range, 3-14 Gy) for studies using photon-based IORT. For protocols which included pre-op EBRT, mean doses were 32.9 Gy (range, 9.2–45 Gy) for studies on IOERT, 9.1 Gy (range 9.1-9.1 Gy) for studies on IOHDR, and 9.2Gy (range, 9.1-9.2 Gy) for studies with a mix of IOERT or IOHDR. The adjuvant EBRT average doses received by patients who underwent IORT ranged from 47.1 to 51.9 Gy.

### Follow-up and outcomes

Follow-up of the selected studies were varied with follow-up periods as short as 0 to as long as 262 months. Average LC, LRC, DFS and OS from the selected studies were 69.28%, 65.63%, 51.88% and 53.01%, respectively.

Acute toxicities reported were wound infection/dehiscence in 1.6%–54% of patients [8, 10, 14, 16, 18, 19, 22, 27, 34, 36–39, 41, 48, 51–53, 55, 59], necrosis or flap failure in 0.7%–23% [14, 16, 18, 23, 26, 28–30, 38, 39, 52, 55, 59], osteonecrosis in 1.1%–8% [20, 22, 36, 38, 39, 41, 44, 45, 50], fistula in 1.5%–20% [8, 10, 14, 16, 18–20, 24–26, 30, 31, 33, 34, 37–39, 41, 44, 46, 52, 53, 59], trismus in 4.5%–24.6% [24, 25, 37], skin/mucosal complications in 2.3%–100% [8, 10, 14–16, 24, 25, 36, 37, 59] and neuropathy in 0.7%–26% [8, 14, 22–27, 36–39, 52, 55, 59]. Other less commonly reported toxicities were carotid/artery rupture (fatal and non-fatal) in 1.1%–5.3% [20, 23, 37, 41, 44, 46, 50, 52, 57] and haematoma in 0.8%–6.3% [10, 14, 30, 31, 39, 59].

Notable late toxicities reported included stenosis or stricture in 1.6%–4.7% [8, 14, 16], infection in 0.8%–16.7% [14, 17, 47, 59], xerostomia in 0.8%–9.3% [8, 14, 47], dysphagia in 26% [13], osteoradionecrosis in 1.6%–17.4% [14, 21, 32] and carotid artery rupture in 2.3%–3.6% [32, 36, 57].

## Discussion

IORT as a treatment modality has already been utilised in reports spanning decades; however, the body of published evidence is primarily taken from single-institution retrospective studies with varied patient populations, prior history of radiation treatment, radiation dose prescribed and modalities of treatment delivery (IOERT, IOHDR, Photon IORT), leading to wide ranges in reported control and toxicity outcomes. Despite the heterogeneity, we can note several insights from the available body of published evidence.

First, in terms of patient selection, IORT has shown LC benefit in patients with high risk of locoregional recurrence such as patients with locally advanced tumours in the primary setting, or those with recurrences following prior failed treatment undergoing salvage interventions. This makes sense radiobiologically as previously discussed by Hilal *et al* [6], since IORT can deliver a reported biological effective dose 1.5–2.5 times higher than standard fractionation. In addition, IORT with or without additional EBRT favourably reduces the over-all treatment time by delivering a significant amount of radiation dose at the time of excision surgery.

Second, in terms of histopathology, IORT has shown its utility not just in SCC – the predominant type of head and neck cancer, but also in other tumour types such as salivary gland malignancies. Regardless of pathologic type, however, we also note that effectiveness is affected by margin status where highest benefit of IORT best seen when done in resections which are margin-negative (R0) or up to microscopic positive (R1), with lesser effect seen for those with macroscopic positive (R2) margins [18, 26, 35, 37, 46, 49, 50, 57].

Third, in terms of technique, although IOERT has been the most widely studied, IOHDR and photon IORT have also been shown to be effective techniques of treatment delivery. We were able to note that the median doses prescribed for each technique in the included studies were 15.6 Gy (4.0–100.0 Gy) for studies using IOERT, 13.4 Gy (5.0–20.0 Gy) for IOHDR, 5.8 Gy (3.0–14.0 Gy) for photon IORT. These findings are consistent with those of Kyrgias *et al* [7] who also discussed a trend over time of lowering IORT dose from a maximum of 20 Gy in order to decrease treatment related toxicity. Aside from innate differences in delivery method and radiation physics properties of each technique, these dose differences must be interpreted with caution since these studies were also done in heterogenous populations – with variances in margin status, history of prior irradiation, as well as use of additional EBRT combined with IORT. Indirect comparisons are problematic; moreover, we did not encounter any trials comparing each of the delivery techniques directly.

Fourth, in terms of outcomes, again with the caveat of significant heterogeneity, we were still able to see LC across publications averaging around 69% and OS averaging 53% at roughly 2–3 years post-IORT treatment follow-up. Toxicity rates were varied and difficult to isolate for IORT exclusively, because these patients often received multi-course as well as multi-modality treatment.

Looking at the available evidence in total, we surmise that IORT for head and neck tumours may find most utility in well selected patients as an immediate boost as part of a reirradiation regimen together with additional EBRT following salvage surgery with good margins in the recurrent setting. This would allow it to leverage its strengths of precise local treatment delivery, theoretical decreased toxicity through the ability to physically move away nearby normal structures, decreased over-all treatment time and immediate high dose delivery to what may be radiobiologically unfavourable tumours due to hypoxia and radiation resistance from prior therapy. Additional EBRT appears to still be necessary to reach appropriate total doses beyond the accepted safe limits of IORT delivery.

Foreseen possible disadvantages to use of IORT include the limited treatment area the intraoperative applicators can cover. This can be overcome by planning for consecutive treatment of multiple adjacent treatment fields, but this would entail a dramatic increase in over-all procedure time. Another barrier to usage is the substantial cost of additional equipment, radiation safety and training which requires both institutional and multi-disciplinary investment and commitment. This can be compensated by leveraging the use of IORT for other tumour subsites particularly for breast, gynaecologic, gastrointestinal and soft tissue cancer. Finally, the greatest barrier to greater acceptance and usage of IORT particularly for the head and neck cancer is the lack of higher level evidence supporting its use. Multi-institutional collaborative trails are needed in order to accrue patients and compare outcomes of IORT with current available standard modalities of care.

## Conclusions

IORT as a treatment modality in head and neck cancer has shown promise with multiple single-institutional studies that have shown benefits in terms of LC with reported reasonable toxicity. However, in order to progress further, similar to that seen in the use of IORT in other anatomical sites, randomized control trials need to be developed comparing IORT with current standards of care in the modern era of intensity modulated radiation therapy and volumetric modulated arc therapy.

## List of abbreviations

DFS, Disease free survival; EBRT, External beam radiation therapy; IOERT, Intraoperative electron radiation therapy; IOHDR, Intraoperative high dose-rate brachytherapy; IORT, Intraoperative radiation therapy; LC, local control; LRC, Locoregional control; OS, Overall survival; SCC, Squamous cell carcinoma.

## Conflicts of interest

The authors declared that they have no competing interests.

## Authors’ contributions

Primary author: Cesar Vincent L Villafuerte III. Contributing authors: Aveline Marie D Ylanan, Harroun Valdimir T Wong, Johanna Patricia A Cañal and Edilberto Joaquin V Fragante Jr.

All authors gave substantial contributions to the conception or design of the work; or the acquisition, analysis or interpretation of data for the work; drafting the work or revising it critically for important intellectual content; and final approval of the version to be published. They agree to be accountable for all aspects of the work in ensuring that questions related to the accuracy or integrity of any part of the work are appropriately investigated and resolved.

## Funding

The authors did not receive any external sources of funding.

## Figures and Tables

**Table 2.** Summary of studies.

	Electron	Brachytherapy	Electron or Brachytherapy	Photon	All studies
Number of studies	27	10	10	5	52
Number of patients	1,476	445	400	68	2,389
Primary	358	117	287	31	793
Recurrent	1,000	328	20	28	1,376
Not reported	118	0	93	9	220
Reported use of prior RT					
No prior RT	408	147	364	16	935
Reirradiation	841	221	33	0	1,095
Not reported	227	77	3	52	359
Average IORT doses^a^	15.6 (4.0–100.0) Gy	13.4 (5.0–20.0) Gy	8.3 (7.5–20.0) Gy	5.8 (3.0–14.0) Gy	
Average EBRT doses^a^					
Previous irradiation	54.5 (0–66) Gy	61.6 (54–70) Gy	34.1 (17.5–50.7) Gy	NA	
Pre IORT EBRT	32.9 (9.2–45) Gy	9.1 (9.1–9.1) Gy	9.2 (9.1–9.2) Gy	NA	
Post IORT EBRT	47.1 (40–54) Gy	51.9 (45–63) Gy	50 (50–50) Gy	50 (50–50) Gy	
Average outcomes^a^					
Follow-up	34.4 (0.1–262.2) months	32.3 (1–133) months	38.7 (0–120.4) months	21.2 (0–66) months	33.5 (0–262.2) months
LC	62.3 (16.7–100)%	63.7 (34–83.3)%	90.6 (70–100)%	89.8 (85–100)%	69.3 (16.7–100)%
LRC	56.4 (21.2–91)%	57.9 (29–100)%	91.8 (88–95)%	81.3 (81.3–81.3)%	65.6 (21.2–100)%
DFS	45.1 (11–70.3)%	46.4 (24–100)%	79.3 (72–92.5)%	68.8 (68.8–68.8)%	51.9 (11–100)%
OS	48 (8–88)%	52.9 (29–100)%	66.1 (48.6–79)%	81.3 (81.3–81.3)%	53 (8–100)%

a In computing summary values for this table, only studies that reported such parameters were considered. To summarise the doses used among the studies, averages of median doses were obtained. Mean doses were used for the few studies that did not provide a median dose. Because follow-up period varied, outcomes reported at the specified median follow-up were used to compute for the average. For studies that did not provide this, outcomes with the longest period were used.

## References

[1] National Comprehensive Cancer Network (2021). Head and Neck Cancers (version 3.2021) NCCN Clinical Practice Guidelines in Oncology.

[2] Machiels JP, René Leemans C, Golusinski W (2020). Squamous cell carcinoma of the oral cavity, larynx, oropharynx and hypopharynx: EHNS–ESMO–ESTRO clinical practice guidelines for diagnosis, treatment and follow-up. Ann Oncol.

[3] Wuthrick EJ, Zhang Q, Machtay M (2015). Institutional clinical trial accrual volume and survival of patients with head and neck cancer. J Clin Oncol.

[4] Chang JH, Wu CC, Yuan KSP (2017). Locoregionally recurrent head and neck squamous cell carcinoma: incidence, survival, prognostic factors, and treatment outcomes. Oncotarget.

[5] Pilar A, Gupta M, Laskar SG (2017). Intraoperative radiotherapy: review of techniques and results. Ecancermedicalscience.

[6] Hilal L, Al Feghali KA, Ramia P (2017). Intraoperative radiation therapy: a promising treatment modality in head and neck cancer. Front Oncol.

[7] Kyrgias G, Hajiioannou J, Tolia M (2016). Intraoperative radiation therapy (IORT) in head and neck cancer: a systematic review. Medicine.

[8] Grecula JC, Schuller DE, Rhoades CA (1999). Intensification regimen 2 for advanced head and neck squamous cell carcinomas. Arch Otolaryngol Head Neck Surg.

[9] Schuller DE, Grecula JC, Gahbauer RA (1997). Intensified regimen for advanced head and neck squamous cell carcinomas. Arch Otolaryngol Head Neck Surg.

[10] Grecula JC, Schuller DE, Smith R (2001). Long-term follow-up on an intensified treatment regimen for advanced resectable head and neck squamous cell carcinomas. Cancer Invest.

[11] Malone JP, Stephens JA, Grecula JC (2004). Disease control, survival, and functional outcome after multimodal treatment for advanced-stage tongue base cancer. Head Neck.

[12] Schuller DE, Grecula JC, Agrawal A (2002). Multimodal intensification therapy for previously untreated advanced resectable squamous cell carcinoma of the oral cavity, oropharynx, or hypopharynx. Cancer.

[13] Ozer E, Grecula JC, Agrawal A (2006). Intensification regimen for advanced-stage resectable hypopharyngeal carcinoma. Arch Otolaryngol Head Neck Surg.

[14] Schuller DE, Ozer E, Agrawal A (2007). Multimodal intensification regimens for advanced, resectable, previously untreated squamous cell cancer of the oral cavity, oropharynx, or hypopharynx. Arch Otolaryngol Head Neck Surg.

[15] Rutkowski T, Wygoda A, Hutnik M (2010). Intraoperative radiotherapy (IORT) with low-energy photons as a boost in patients with early-stage oral cancer with the indications for postoperative radiotherapy: treatment feasibility and preliminary results. Strahlenther Onkol.

[16] Schmitt T, Prades JM, Favrel V (1997). IORT for locally advanced oropharyngeal carcinomas with major extension to the base of the tongue: 5-year results of a prospective study. Front Radiat Ther Oncol.

[17] Marr BP, Abramson DH, Cohen GN (2015). Intraoperative high-dose rate of radioactive phosphorus 32 brachytherapy for diffuse recalcitrant conjunctival neoplasms: a retrospective case series and report of toxicity. JAMA Ophthalmol.

[18] Schleicher UM, Phonias C, Spaeth J (2001). Intraoperative radiotherapy for pre-irradiated head and neck cancer. Radiother Oncol.

[19] Nag S, Schuller DE, Martinez-Monge R (1998). Intraoperative electron beam radiotherapy for previously irradiated advanced head and neck malignancies. Int J Radiat Oncol Biol Phys.

[20] Rate WR, Garrett P, Pugh N (1991). Intraoperative radiation therapy for recurrent head and neck cancer. Cancer.

[21] Nag S, Schuller DE, Rodríguez-Villalba S (1999). Intraoperative high dose rate brachytherapy can be used to salvage patients with previously irradiated head and neck recurrences. Rev Med Univ Navarra.

[22] Stewart R, Hu K, Berlach D (2015). High-dose-rate intraoperative radiotherapy combined with neck dissection to salvage isolated cervical nodal recurrences. Brachytherapy.

[23] Scala LM, Hu K, Urken ML (2013). Intraoperative high-dose-rate radiotherapy in the management of locoregionally recurrent head and neck cancer. Head Neck.

[24] Teckie S, Scala LM, Ho F (2013). High-dose-rate intraoperative brachytherapy and radical surgical resection in the management of recurrent head-and-neck cancer. Brachytherapy.

[25] Perry DJ, Chan K, Wolden S (2010). High-dose-rate intraoperative radiation therapy for recurrent head-and-neck cancer. Int J Radiat Oncol Biol Phys.

[26] Chen AM, Bucci MK, Singer MI (2007). Intraoperative radiation therapy for recurrent head-and-neck cancer: the UCSF experience. Int J Radiat Oncol Biol Phys.

[27] Chen AM, Garcia J, Bucci MK (2008). Recurrent salivary gland carcinomas treated by surgery with or without intraoperative radiation therapy. Head Neck.

[28] Cristalli G, Mercante G, Marucci L (2016). Radioterapia intraoperatoria nei tumori maligni avanzati estesi all’orecchio medio: valutazione da uno studio retrospettivo. Acta Otorhinolaryngol Ital.

[29] Cristalli G, Manciocco V, Pichi B (2009). Treatment and outcome of advanced external auditory canal and middle ear squamous cell carcinoma. J Craniofac Surg.

[30] Marucci L, Pichi B, Iaccarino G (2008). Intraoperative radiation therapy as an “‘Early Boost’” in locally advanced head and neck cancer: preliminary results of a feasibility study. Head Neck.

[31] Nag S, Schuller D, Pak V (1997). IORT using electron beam or HDR brachytherapy for previously unirradiated head and neck cancers. Front Radiat Ther Oncolo.

[32] Toita T, Nakano M, Takizawa Y (1994). Intraoperative radiation therapy (IORT) for head and neck cancer. Int J Radiati Oncol Biol Phys.

[33] Wolf G, Geyer E, Langsteger W (1995). Intraoperative radiation therapy in advanced thyroid cancer. Eur J Surg Oncol.

[34] Spaeth J, Andreopoulos D, Unger T (1997). Intra-operative radiotherapy - 5 years of experience in the palliative treatment of recurrent and advanced head and neck cancers. Oncology (Switzerland).

[35] Martínez-Monge R, Azinovic I, Alcalde J (1997). IORT in the management of locally advanced or recurrent head and neck cancer. Front Radiat Ther Oncol.

[36] Coleman CW, Roach M, Ling SM (1997). Adjuvant electron-beam IORT in high-risk head and neck cancer patients. Front Radiat Ther Oncol.

[37] Pinheiro AD, Foote RL, McCaffrey TV (2003). Intraoperative radiotherapy for head and neck and skull base cancer. Head Neck.

[38] Zeidan YH, Yeh A, Weed D (2011). Intraoperative radiation therapy for advanced cervical metastasis: a single institution experience. Radiat Oncol.

[39] Zeidan YH, Shiue K, Weed D (2012). Intraoperative radiotherapy for parotid cancer: a single-institution experience. Int J Radiat Oncol Biol Phys.

[40] Novikov VA, Gribova OV, Vasiljev RV (2017). Intraoperative radiotherapy in combined treatment of sinonasal malignant tumors. AIP Conference Proceedings.

[41] Wald P, Grecula J, Walston S (2019). Intraoperative electron beam radiotherapy for locoregionally persistent or recurrent head and neck cancer. Head Neck.

[42] Kopp M, Meco S, Oberascher G (2009). Intraoperative radiotherapy in advanced tumours of the anterior skull base. The results of 30 patients over a eight year period. Radiother Oncol.

[43] Kopp M (2020). Local tumor control and long-term overall survival in 51 patients with anterior skull base tumors treated with intraoperative radiation therapy. Int J Radiat Oncol Biol Phys.

[44] Freeman SB, Hamaker RC, Singer MI (1990). Intraoperative radiotherapy of head and neck cancer. Arch Otolaryngol Head Neck Surg.

[45] Freeman SB, Hamaker RC, Singer MI (1991). Intraoperative radiotherapy of skull base cancer. Laryngoscope.

[46] Freeman SB, Hamaker RC, Huntley TC (1995). Management of advanced cervical metastasis using intraoperative radiotherapy. Laryngoscope.

[47] Nag S, Schuller D, Pak V (1996). Pilot study of intraoperative high dose rate brachytherapy for head and neck cancer. Radiother Oncol.

[48] Nag S, Tippin D, Grecula J (2004). Intraoperative high-dose-rate brachytherapy for paranasal sinus tumors. Int J Radiat Oncol Biol Phys.

[49] Nag S, Koc M, Schuller DE (2005). Intraoperative single fraction high-dose-rate brachytherapy for head and neck cancers. Brachytherapy.

[50] Hu K, Scala M, Rao M (2010). High-dose-rate intraoperative radiation therapy for the salvage treatment of head and neck cancer. Int J Radiat Oncol Biol Phys.

[51] Haller JR, Mountain RE, Schuller DE (1996). Mortality and morbidity with intraoperative radiotherapy for head and neck cancer. Am J Otolaryngol Head Neck Med Surg.

[52] Most MD, Allori AC, Hu K (2008). Feasibility of flap reconstruction in conjunction with intraoperative radiation therapy for advanced and recurrent head and neck cancer. Laryngoscope.

[53] Yi PQ, Nie FF, Fan YB (2017). Intraoperative radiotherapy for the treatment of thyroid cancer: a pilot study. Oncotarget.

[54] Emami B, Borrowdale R, Choi M (2016). High dose-low energy intraoperative radiotherapy in the treatment of malignant H&N tumors. Radiother Oncol.

[55] Emami B, Borrowdale RW, Sethi A (2017). Intraoperative radiation therapy in head and neck cancers. Int J Radiat Oncol Biol Phys.

[56] Majercakova K, Isern Verdum J, Alegre M (2014). “Intraoperative-like” single dose X-ray radiotherapy in squamous cell skin carcinoma in elderly patients. Radiother Oncol.

[57] Garrett P, Pugh N, Ross D (1987). Intraoperative radiation therapy for advanced or recurrent head and neck cancer. Int J Radiat Oncol Biol Phys.

[58] Akrami M, Nasrollahi H, Vahabi M (2020). Intraoperative radiation therapy in non-breast cancer patients: a report of 26 cases from Shiraz, south of Iran. Med J Islam Repub Iran.

[59] Ozer E, Grecula JC, Agrawal A (2006). Long-term results of a multimodal intensification regimen for previously untreated advanced resectable squamous cell cancer of the oral cavity, oropharynx, or hypopharynx. Laryngoscope.

